# Enhanced expression of PD-L1 in non-muscle-invasive bladder cancer after treatment with Bacillus Calmette-Guerin

**DOI:** 10.18632/oncotarget.26122

**Published:** 2018-09-25

**Authors:** Akihito Hashizume, Susumu Umemoto, Tomoyuki Yokose, Yoshiyasu Nakamura, Mitsuyo Yoshihara, Kahori Shoji, Satoshi Wada, Yohei Miyagi, Takeshi Kishida, Tetsuro Sasada

**Affiliations:** ^1^ Department of Urology, Kanagawa Cancer Center, Yokohama, Japan; ^2^ Department of Pathology, Kanagawa Cancer Center, Yokohama, Japan; ^3^ Kanagawa Cancer Center Research Institute, Yokohama, Japan; ^4^ Cancer Vaccine Center, Kanagawa Cancer Center, Yokohama, Japan

**Keywords:** PD-L1, non-muscle-invasive bladder cancer, Bacillus Calmette-Guerin, CD8, immune checkpoint

## Abstract

Immune checkpoint molecules, such as PD-1/PD-L1, are reported to be closely associated with suppression of antitumor immunity, and their inhibitors have been used to treat various cancers including bladder cancer. However, there have been only a few studies investigating the effects of Bacillus Calmette-Guerin (BCG) administration on expression of the immune checkpoint molecules in bladder cancer. The current study examined the expression of PD-L1 and PD-L2 before and after BCG in non-muscle-invasive bladder cancer (NMIBC) patients. Tissue microarrays of 22 BCG-resistant NMIBC patients were stained by immunohistochemistry with antibodies against PD-L1, PD-L2, and CD8, and were compared between before and after BCG. The expression levels of PD-L1, but not of PD-L2, were significantly increased after BCG treatment on tumor cells (p < 0.001) and tumor-infiltrating inflammatory cells (p = 0.030) within tumor tissues, as well as on inflammatory cells within non-tumor normal tissues (p = 0.003). Although CD8^+^ T cells were significantly increased within tumor tissues (p = 0.005) and non-tumor normal tissues (p = 0.007) after BCG treatment, they might be not effective for anti-tumor immunity. This study demonstrated for the first time that expression of PD-L1, which might contribute to the immune escape mechanism, was enhanced on tumor tissue after BCG treatment in BCG-resistant NMIBC patients. Our finding thus propose that immunotherapy with anti-PD-1/PD-L1 antibodies could be feasible as combination treatment with BCG or as secondary treatment at relapse after BCG in NMIBC patients.

## INTRODUCTION

Non-muscle-invasive bladder cancer (NMIBC) comprises approximately 70% of all untreated bladder cancers. Transurethral resection of bladder tumor (TURBT) is typically selected as the first line of treatment for NMIBC [1]. Intravesical administration of Bacillus Calmette-Guerin (BCG) after TURBT is performed for those patients with carcinoma in situ (CIS) that cannot be completely resected by TURBT, as well as to prevent postoperative recurrence [1–4]. This therapy is a standard procedure recommended by national and international guidelines for high-risk NMIBC, and it has been shown to reduce recurrence and disease progression. According to the European Association of Urology (EAU) guideline, high-risk NMIBC is defined as those that meet any of the following criteria: T1, G3 (high grade), concurrent CIS, or multiple, recurrent, and large (> 3 cm) TaG1G2/low grade tumors. Since high-risk NMIBC often leads to not only recurrence in the bladder, but also invasion into the muscle and/or dissemination into the upper urinary tract, intravesical therapy with BCG has been employed to prevent tumor recurrence and progression and preserve the bladder. Although some patients show no recurrence after BCG therapy, others fail and require additional treatments such as radical cystectomy. BCG is thought to elicit antitumor response by activating immune cells in the bladder wall [2], but the detailed immunological changes in tumor microenvironment after BCG remain unknown.

Immune checkpoint molecules, such as PD-1/PD-L1, are reported to be closely associated with suppression of antitumor immunity [5, 6], and their inhibitors have been used to treat various cancers including bladder cancer [7–12]. However, there have been only a few studies investigating the expression of molecules involved in the regulation of immune checkpoint pathway before and after BCG therapy, which elicits antitumor immune response [13, 14]. In the present study, we examined the expression of immune checkpoint molecules, including PD-L1 and PD-L2, and CD8 T cell density before and after BCG administration in NMIBC patients who showed resistance to BCG therapy.

## RESULTS

### Increase in PD-L1 expression after BCG treatment in BCG-resistant patients

**Figure 1:**
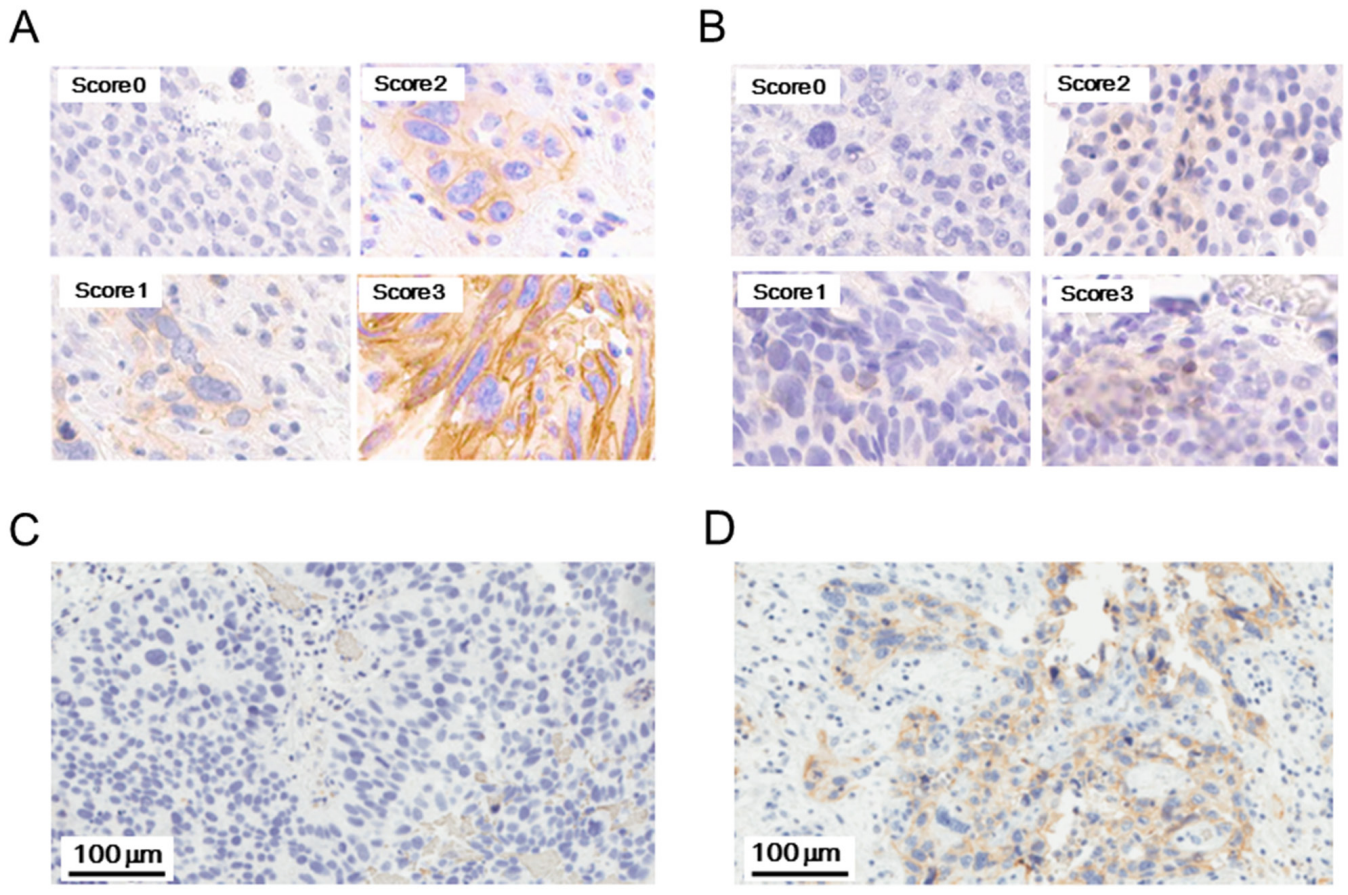
Representative staining patterns of PD-L1 and PD-L2 Staining intensity of PD-L1 **(A)** and PD-L2 **(B)** was categorized into negative (0), weak (1), intermediate (2), and strong (3). The strongest intensity within a sample was selected as intensity score (IS) of the sample. Representative PD-L1 staining images in tumor tissues before **(C)** and after **(D)** BCG treatment.

Figure 1A shows representative PD-L1 immunohistochemistry (IHC) staining patterns in tumor specimens. Immunostaining of PD-L1 was observed in the membrane and/or cytoplasm of the tumor cells and/or stromal inflammatory cells. Figure 1C and 1D show representative PD-L1 staining images in tumor tissues before and after BCG treatment, respectively.

The clinicopathological characteristics of the 22 NMIBC patients, who showed resistance to BCG therapy, are shown in Table 1. Before BCG treatment, PD-L1 expression was positive in tumor cells and tumor-infiltrating inflammatory cells within tumor tissues from 2 (9%) and 11 (50%) of 22 BCG-resistant patients, respectively (Table 2). In non-tumor normal tissues that were available in 20 patients, inflammatory cells were positive for PD-L1 expression in 10 patients (50%). There was no statistically significant correlation in PD-L1 expression among tumor cells and inflammatory cells in tumor tissues and inflammatory cells in non-tumor normal tissues (data not shown, by Spearman’s rank correlation coefficient).

**Table 1:** Patients’ clinicopathological characteristics

Characteristics	BCG-resistant (n = 22)	Recurrence-free (n = 8)
Sex		
Male	18	6
Female	4	2
Age (years)		
median (range)	68.5 (27 - 84)	80 (72 - 86)
T stage		
pTa	8	0
pT1	4	7
pTis	6	1
pTa + pTis	3	0
unknown	1	0
Tumor grade		
Grade 2	5	1
Grade 3	17	7
BCG reagent		
ImmunoCyst (81mg)	3	2
Immunobladder (40mg)	7	1
Immunobladder (80mg)	10	5
unknown	2	0
Number of BCG administration		
median (range)	8 (6 - 12)	8 (6 – 10)
BCG resistance		
refractory	8	NA^a^
relapsing	14	NA
Time from BCG therapy to recurrence (months)		
median (range)	23 (3 – 120)	NA
Follow-up period from BCG therapy (months)		
median (range)	NA	65.5 (35 – 82)

^a^NA: not assessed.

**Table 2:** PD-L1 expression on tumor cells, tumor-infiltrating inflammatory cells, and inflammatory cells in normal tissue before and after BCG therapy in the BCG-resistant patients

Patient number	Tumor cells		Tumor-infiltrating inflammatory cells		Inflammatory cells in normal tissue	
	Before	After	Before	After	Before	After
1	0^a^	0	4	2	4	0
2	0	3	0	5	0	NA^b^
3	0	3	3	4	0	2
4	0	6	4	2	3	5
5	0	2	0	3	NA	0
6	3	2	0	4	4	4
7	0	5	4	6	0	4
8	0	4	0	5	0	4
9	0	3	0	4	2	0
10	0	0	3	0	3	4
11	0	4	0	4	0	4
12	0	3	0	5	0	4
13	0	3	4	4	0	4
14	0	4	0	4	0	4
15	0	2	0	5	NA	4
16	0	0	3	3	2	5
17	0	3	0	4	0	5
18	0	5	0	0	3	NA
19	0	0	3	3	2	4
20	0	0	4	0	3	5
21	2	0	4	0	3	5
22	0	0	3	4	0	3

^a^sum of intensity score (IS) and proportion score (PS) was counted.

^b^NA: not assessed due to lack of available tissues.

**Figure 2:**
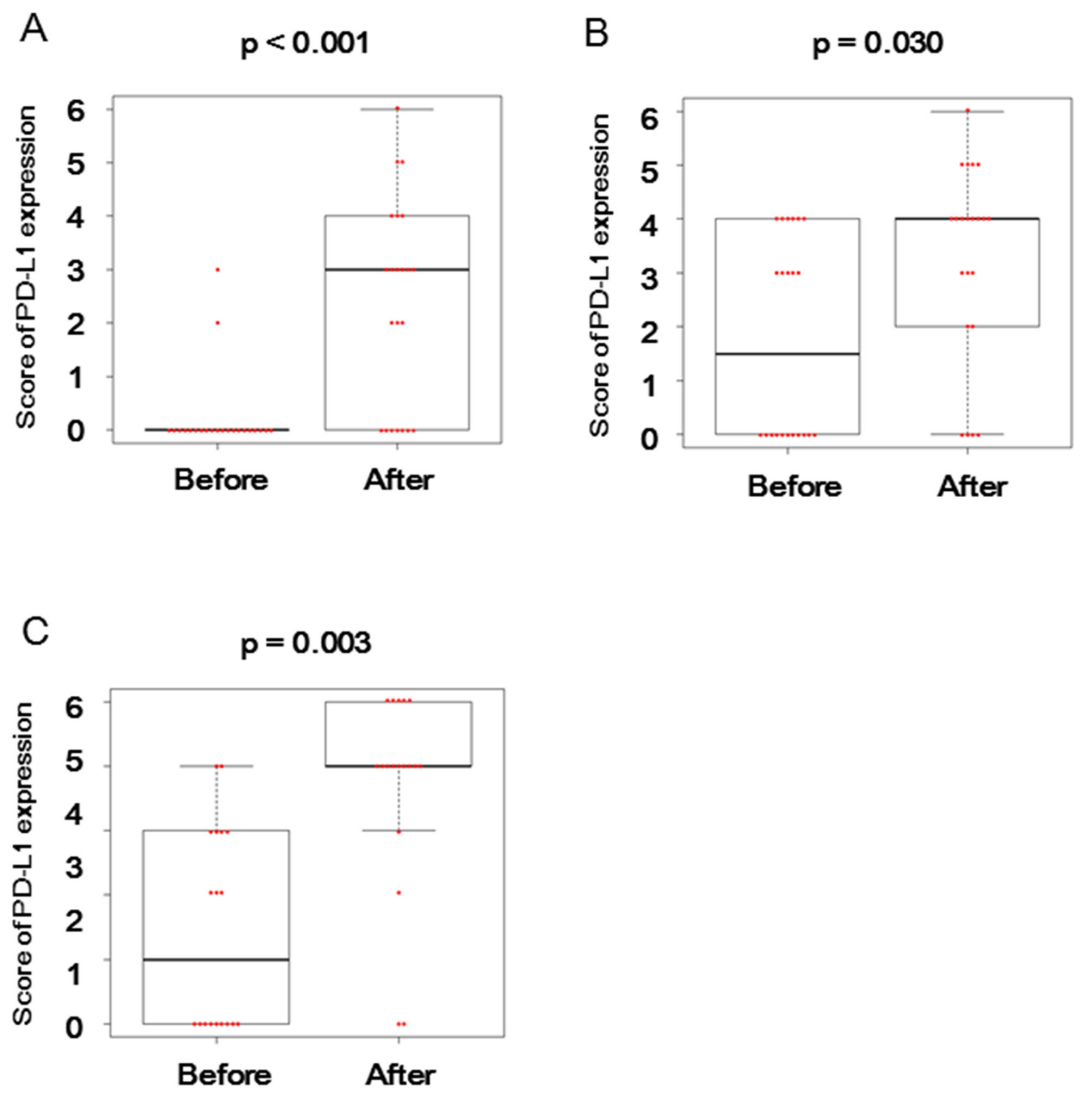
PD-L1 expression before and after BCG treatment in BCG-resistant patients Distributions of PDL-1 expression scores before and after BCG were shown in tumor cells **(A)** and tumor-infiltrating inflammatory cells **(B)** within tumor tissues or in inflammatory cells within non-tumor normal tissues **(C)** from the BCG-resistant patients. The horizontal line in the middle of each box indicates the median, whereas the top and bottom borders of the box mark the 75^th^ and 25^th^ percentiles, respectively. The upper whisker is the 75^th^ percentile + (1.5 × interquartile range) and the lower whisker is the 25^th^ percentile − (1.5 × interquartile range). The two-sided Wilcoxon test was used for their comparison.

After BCG treatment, PD-L1 expression on tumor cells became positive in 14 (70%) of 20 patients, who showed no PD-L1 expression before treatment (Table 2). In contrast, the PD-L1 levels were decreased in 2 patients with PD-L1-positive tumor cells before treatment (Table 2). As shown in Figure 2A, PD-L1 expression on tumor cells was significantly enhanced after BCG treatment (p < 0.001). In addition, PD-L1 expression levels were increased in tumor-infiltrating inflammatory cells from 13 (59%) patients, but were decreased in 5 (23%) patients after BCG treatment (Table 2). As shown in Figure 2B, there was also statistically significant increase in PD-L1 expression on tumor-infiltrating inflammatory cells after treatment (p = 0.030).

Similarly, after BCG treatment, PD-L1 expression levels were increased on inflammatory cells in non-tumor normal tissues from 15 (83%) of 18 patients, but were decreased in 2 (11%) patients (Table 2). A statistically significant increase (p = 0.003) was also observed in PD-L1 expression on inflammatory cells in non-tumor normal tissues after treatment (Figure 2C).

### Increase in T cell infiltration after BCG treatment in BCG-resistant patients

**Figure 3:**
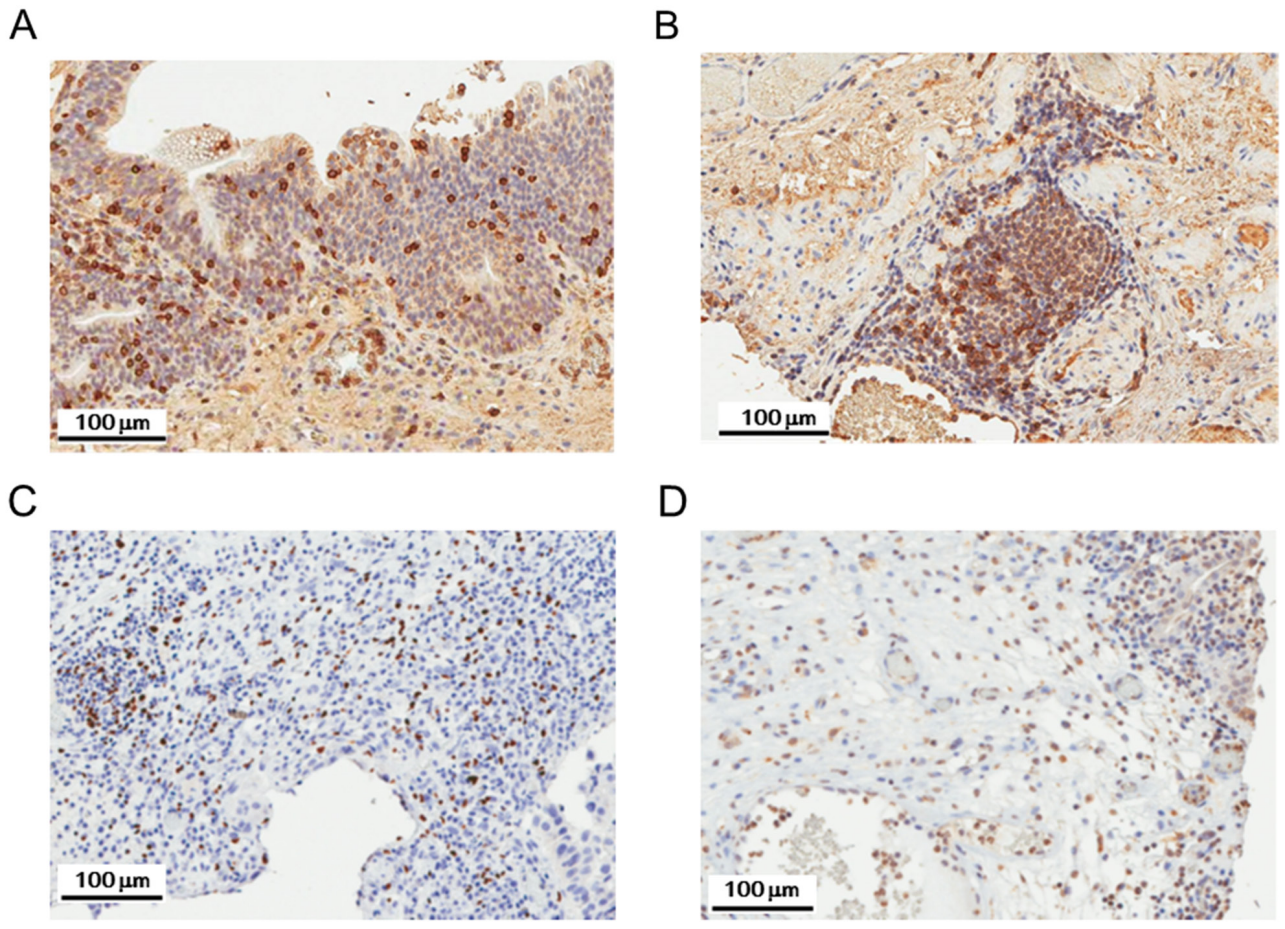
Representative staining patterns of CD8, FOXP3, and CD69 Representative CD8^+^ T cell staining pattern within tumor tissue **(A)** and focal CD8^+^ T cell aggregates within non-tumor normal tissue **(B)**. Representative FOXP3 **(C)** and CD69 **(D)** staining images in tumor tissues.

**Figure 4:**
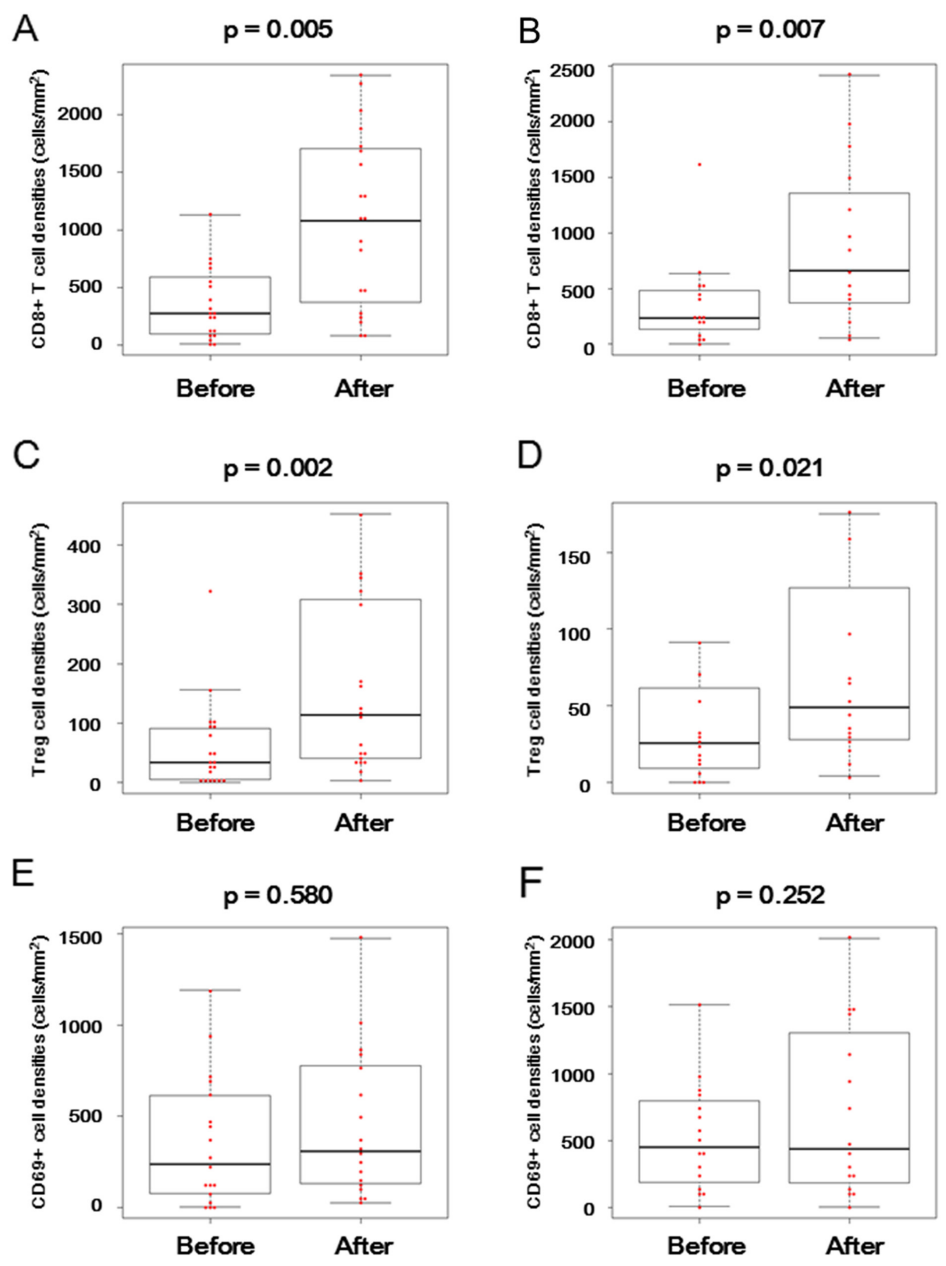
CD8^+^ T cell, FOXP3^+^ regulatory T cell, and CD69^+^ activated cell infiltration before and after BCG treatment in BCG-resistant patients Distributions of CD8^+^ T cell density (cells/mm^2^) within tumor tissues **(A)** or non-tumor normal tissues **(B)**, FOXP3^+^ regulatory T cell density (cells/mm^2^) within tumor tissues **(C)** or non-tumor normal tissues **(D)**, and CD69^+^ activated cell density (cells/mm^2^) within tumor tissues **(E)** or non-tumor normal tissues **(F)** were shown before and after BCG in the BCG-resistant patients. The horizontal line in the middle of each box indicates the median, whereas the top and bottom borders of the box mark the 75^th^ and 25^th^ percentiles, respectively. The upper whisker is the 75^th^ percentile + (1.5 × interquartile range) and the lower whisker is the 25^th^ percentile − (1.5 × interquartile range). The two-sided Wilcoxon test was used for their comparison.

Figure 3A and 3B show representative CD8 T cell staining patterns by IHC in tumor tissue and non-tumor normal tissue specimens. After BCG treatment, CD8^+^ T cell density (cells/mm^2^) within tumor tissues was increased in 15 (75%) of 20 patients, whose tumor tissues were available for this analysis (Figure 4A and Table 3). There was statistically significant difference in CD8^+^ T cell density in tumor tissues between before and after BCG treatment (p = 0.005). In addition, the prevalence of focal CD8^+^ T cell aggregates within tumor tissues was also increased after BCG treatment; they were observed only in 4 (20%) of 20 patients before BCG, but were more frequently observed after BCG treatment [10 (50%) of 20 patients] (Table 3). Similarly, CD8^+^ T cell density (cells/mm^2^) within non-tumor normal tissues were also significantly increased after BCG treatment (p = 0.007) (Figure 4B).

**Table 3:** CD8^+^ T cell density on tumor cells and non-tumor normal tissue before and after BCG therapy in the BCG-resistant patients

Patient number	Tumor tissue^a^		Non-tumor normal tissue^a^	
	Before	After	Before	After
1	2536.0^c^	2052.0^c^	521.0	55.2
2	403.6	191.8	715.5	NA^b^
3	511.2	242.3^c^	635.3	660.0^c^
4	131.6	472.0	255.0	848.0
5	9.4	269.6^c^	NA	68.1
6	281.2	1708.0^c^	193.8	2420.0^c^
7	122.4	2344.0^c^	83.2	441.9^c^
8	325.4^c^	1084.0	227.5	516.0^c^
9	246.1	1080.0	525.2	988.0
10	690.0	828.0^c^	443.9^c^	1212.0
11	728.4	475.3	184.1	337.7
12	267.7	1888.0^c^	42.1	210.6^c^
13	230.1	1308.0	634.5	NA
14	NA	780.0	407.0	406.4^c^
15	NA	220.0^c^	NA	1520.0^c^
16	650.9^c^	1308.0^c^	233.2	94.7
17	56.7	2272.0	52.4	1508.0
18	76.8	84.9	2620.0^c^	NA
19	1132.0	1548.0^c^	NA	204.0
20	533.0^c^	81.0	NA	2016.0^c^
21	21.1	912.0	1624.0^c^	1980.0^c^
22	66.4	1704.0	1.2	1784.0^c^

^a^CD8^+^ T cell density (cells/mm^2^) was assessed.

^b^NA: not assessed due to lack of available tissues.

^c^Focal CD8^+^ T cell aggregates were detected within tissues.

Figure 3C and 3D show representative staining patterns of a regulatory T (Treg) cell marker, FOXP3, and an early activation marker of lymphocytes, CD69, by IHC in tumor tissue specimens. As shown in Figure 4C and 4D, FOXP3^+^ Treg cell density (cells/mm^2^) was significantly increased within tumor (p = 0.002) and non-tumor normal (p = 0.021) tissues after BCG treatment. In contrast, there was no significant difference in CD69^+^ cell density (cells/mm^2^) on tumor (p = 0.580) or non-tumor normal (p = 0.252) tissues between before and after BCG (Figure 4E and 4F).

### PD-L2 expression before and after BCG treatment in BCG-resistant patients

Figure 1B shows representative PD-L2 IHC staining patterns in tumor specimens. As shown in Table 4, before BCG treatment, PD-L2 expression was positive in tumor cells and tumor-infiltrating inflammatory cells within tumor tissues from 2 (10%) and 1 (5%) of 21 BCG-resistant patients, respectively. In non-tumor normal tissues that were available in 20 patients, inflammatory cells positive for PD-L2 expression were detected in 2 patients (10%). After BCG treatment, PD-L2 expression in tumor cells was increased or decreased in 1 (5%) and 1 (5%) of 19 patients, respectively. The PD-L2 levels on tumor-infiltrating inflammatory cells were increased or decreased after treatment in 2 (10%) and 1 (5%) of 19 patients, respectively, whereas those on inflammatory cells within non-tumor normal tissues were increased or decreased after treatment in 1 (6%) and 2 (11%) of 18 patients, respectively. No statistically significant changes in PD-L2 expression were observed in tumor tissues or non-tumor normal tissues after BCG treatment (data not shown).

**Table 4:** PD-L2 expression on tumor cells, tumor-infiltrating inflammatory cells, and inflammatory cells in normal tissue before and after BCG therapy in the BCG-resistant patients

Patient number	Tumor cells		Tumor-infiltrating inflammatory cells		Inflammatory cells in normal tissue	
	Before	After	Before	After	Before	After
1	4^a^	0	4	0	0	0
2	NA^b^	0	NA	0	0	NA
3	0	0	0	0	3	0
4	0	0	0	0	0	0
5	0	0	0	0	NA	0
6	0	0	0	0	3	0
7	0	0	0	3	0	0
8	0	0	0	0	0	0
9	0	0	0	0	0	0
10	0	0	0	0	0	0
11	0	0	0	0	0	0
12	0	0	0	4	0	4
13	0	0	0	0	0	0
14	0	0	0	0	0	0
15	0	3	0	0	NA	0
16	0	0	0	0	0	0
17	0	0	0	0	0	0
18	4	NA	0	NA	0	NA
19	0	0	0	0	0	0
20	0	NA	0	NA	0	0
21	0	0	0	0	0	0
22	0	0	0	0	0	0

^a^sum of intensity score (IS) and proportion score (PS) was counted.

^b^NA: not assessed due to lack of available tissues.

### PD-L1 expression before and after BCG treatment in recurrence-free patients

The clinicopathological characteristics of the 8 NMIBC patients, who showed no recurrence or tumor progression after BCG therapy, are shown in Table 1. Expression of PD-L1 on tumor cells and tumor-infiltrating inflammatory cells within tumor tissues and on inflammatory cells within non-tumor normal tissues were detected in 1 (13%), 7 (88%), and 4 (50%) of 8 recurrence-free patients before BCG therapy, respectively (Table 5). Following BCG therapy, PD-L1 expression levels were increased on inflammatory cells in non-tumor normal tissues in 7 (88%) of 8 patients (Table 5). There was statistically significant increase (p = 0.016) in PD-L1 expression on inflammatory cells after BCG treatment.

**Table 5:** PD-L1 expression on tumor cells, tumor-infiltrating inflammatory cells, and inflammatory cells in normal tissue before and after BCG therapy in the recurrence-free patients

Patient number	Tumor cells		Tumor-infiltrating inflammatory cells		Inflammatory cells in normal tissue	
	Before	After	Before	After	Before	After
1	0^a^	NA^b^	0	NA	0	0
2	0	NA	6	NA	5	6
3	0	NA	5	NA	0	6
4	0	NA	7	NA	0	7
5	0	NA	6	NA	4	6
6	8	NA	5	NA	0	7
7	0	NA	4	NA	3	7
8	0	NA	4	NA	3	8

^a^sum of intensity score (IS) and proportion score (PS) was counted.

^b^NA: not assessed due to lack of available tissues.

## DISCUSSION

In the current study, we examined and compared the PD-L1 expression levels in NMIBC patients before and after BCG treatment. Our result revealed that PD-L1 expression in tumor tissues and non-tumor normal tissues was significantly increased after BCG treatment. Although several studies have demonstrated prognostic significance of PD-L1 expression on tumor cells and/or immune cells in bladder cancer [15, 16], there have been only a few reports regarding the effects of BCG treatment on PD-L1 expression in bladder cancer [13, 14]. Inman et al demonstrated that PD-L1 expression was increased in BCG-induced granulomas in the bladder after BCG immunotherapy; the majority of NMIBC patients (12 of 16, 75%) that failed BCG treatment exhibited diffuse and intense PD-L1 expression within the BCG-induced granulomas found in proximity to their recurrent tumors [13]. However, in contrast to our finding, they reported that only 2 of the 16 BCG-refractory NMIBC patients represented weak PD-L1 expression on tumor cells themselves after BCG treatment. The discrepancy between our result and theirs could possibly be explained by the different procedure for IHC staining. For example, we used the Ab clone E1L3N for PD-L1 staining, whereas they used a different clone 5H1, which was reported to show weaker staining pattern by IHC than the E1L3N clone [17]. Alternatively, the differences in study protocols, such as BCG reagents, frequency and/or interval of BCG administration, and timing of tissue sampling, might have caused the discrepancy, because PD-L1 expression levels on tumor cells are known to dramatically change from time to time, depending on their microenvironment [6]. Bellmut et al. also reported no association between prior adjuvant BCG exposure and PD-L1 expression on tumor cells and tumor infiltrating mononuclear cells in 69 urothelial carcinoma, but the detailed information on the patients’ clinicopathological characteristics were not shown [14].

To our knowledge, this is the first report showing that PD-L1 expression on tumor tissues was significantly increased after BCG treatment. Although BCG treatment was also shown to increase CD8^+^ T cell density and/or focal CD8^+^ T cell aggregates within tumor tissues in most of the BCG-resistant patients in this study, they could not control tumor growth. It might thus be possible that enhanced expression of PD-L1 on tumor tissues after BCG treatment contributed to the mechanism by which bladder cancer can escape from the cell-killing effects of tumor-specific immune cells. Since unfortunately, we had only formalin-fixed, paraffin-embedded (FFPE) tissue samples, it was difficult to confirm our results by other methods, such as Western blot and quantitative PCR analyses, in this study. Nevertheless, a recent paper demonstrated a high correlation between mRNA expression measured by quantitative PCR and protein expression measured by IHC with the same anti-PD-L1 monoclonal antibody (mAb; clone E1L3N) in bladder cancer [18]. Further investigations would be recommended to confirm the current results by other analytical methods in future prospective studies.

Recently, five immune checkpoint inhibitors, including nivolumab, pembrolizumab, atezolizumab, durvalumab and avelumab, have been approved by the US Food and Drug Administration (FDA) for the management of locally advanced and metastatic urothelial cancer [7–12]. All of these five immune checkpoint inhibitors have been FDA-approved as second-line therapy, and atezolizumab and pembrolizumab have also been for first-line therapy in cisplatin-ineligible patients [7–12]. In contrast, intravesical administration of BCG is performed after TURBT for high-risk patients with NMIBC that cannot be completely resected by TURBT, as well as to prevent postoperative recurrence [1–4]. Therefore, the indication for clinical use of immune checkpoint inhibitors is currently different from that of BCG. Although some patients show no recurrence after BCG therapy, others fail and require additional treatments such as radical cystectomy. Considering the possibility that BCG-induced PD-L1 overexpression on tumor tissues might contribute to the immune escape mechanism in BCG-resistant NMIBC patients, PD-1/PD-L1 blockade therapy may be clinically effective in combination with BCG as adjuvant therapy or as secondary therapy at relapse after BCG. Indeed, several clinical trials of anti-PD-1/PD-L1 mAb in combination with BCG or after BCG failure in NMIBC patients (NCT03106610, NCT02625961, NCT02324582, NCT03258593) are currently ongoing and their results are awaited.

Cellular immunity via activated tumor-specific cytotoxic T cells has been thought to play an important role in anti-tumor effects of BCG [2, 19]. As expected, in the current study, BCG treatment increased CD8^+^ T cells within tumor tissues in most of the bladder cancer patients. Since activated CD8^+^ T cells can produce IFN-γ, which is reported to induce PD-L1 expression in surrounding tumor and inflammatory cells [6], the higher PD-L1 expression after BCG might be at least in part explained by the accelerated infiltration of CD8^+^ T cells in the BCG-treated patients. BCG immunotherapy was also shown to shift the Th2 to Th1 type immune responses that might contribute to its anti-tumor activity [20, 21]. It thus might be possible that BCG-induced Th1 type immunity enhanced INF-γ production and subsequent PD-L1 expression on surrounding tumor cells and inflammatory cells.

Since CD69^+^ lymphocytes on tissue sections did not increase after BCG, acute inflammation caused by BCG had subsided at the time of tissue sampling. However, counter-regulatory and immunosuppressive mechanisms might be activated as a result of chronic inflammation caused by BCG. Indeed, FOXP3^+^ Treg cells were significantly increased within tumor and non-tumor normal tissues after BCG. Previous studies showed that higher FOXP3^+^ Treg cell counts were associated with shorter recurrence-free survival or BCG failure in NMIBC patients treated with BCG [22, 23]. Therefore, it might be possible that increased FOXP3^+^ Treg cells after BCG treatment might be associated with BCG failure in NMIBC patients.

The current study demonstrated that enhanced PD-L1 expression on inflammatory cells after BCG administration was observed not only in BCG-resistant NMIBC patients but also in recurrence-free patients. It might be hypothesized that in recurrence-free patients, tumor-specific cytotoxic T cells had already controlled tumor growth before adaptive immune escape mechanism mediated by PD-1/PD-L1 pathway was developed and ready to function. Alternatively, the enhanced PD-L1 expression on tumor or inflammatory cells might be simply the result of BCG treatment, but does not contribute to tumor immune escape after BCG. Further studies, such as clinical trials to demonstrate synergistic effects of BCG and anti-PD-1/PD-L1 blockers, would be required to clarify the exact roles of enhanced PD-1/PD-L1 expression after BCG.

We also examined expression levels of PD-L2, another ligand of PD-1, in NMIBC patients before and after BCG treatment. Consistent with the previous report [18], PD-L2 expression on tumor tissues was detected in only a limited population of NMIBC patients. In addition, in contrast to PD-L1, PD-L2 expression levels were not substantially affected by BCG treatment. These results suggested that PD-L2 on tumor tissues might not play a major role in the immune escape mechanism in bladder cancer.

In summary, we have demonstrated for the first time that expression of PD-L1, which might contribute to the immune escape mechanism, was enhanced on tumor tissues after BCG treatment in BCG-resistant NMIBC patients. We thus propose that immunotherapy with anti-PD-1/PD-L1 mAb could be feasible as combination treatment with BCG or as secondary treatment at relapse after BCG in NMIBC patients. Nevertheless, the current pilot study has limitations. One of the major drawbacks is the retrospective character with a limited number of patients. Since the sample size of this study was small, our findings should be considered exploratory and hypothesis-generating in nature. Therefore, future investigations and validations in prospective studies with a larger patient sample size would be required.

## MATERIALS AND METHODS

### Patients

Patients who were diagnosed with NMIBC and underwent BCG therapy at the Kanagawa Cancer Center between 2013 and 2015 were retrospectively identified. Among 42 patients who showed resistance to BCG therapy, 22 patients whose pathological specimens were available prior to and following BCG were included in this study. In addition, 8 NMIBC patients without recurrence or tumor progression after BCG therapy, whose specimens were available prior to and following the treatment, during the same study period were also examined. For the intravesical BCG therapy, patients received ImmuCyst (Connaught strain; Sanofi, Paris, France) or Immunobladder (BCG Tokyo 172 strain; Japan BCG Laboratory, Tokyo, Japan). This study was approved by the Institutional Review Board of Kanagawa Cancer Center. Informed consents for the study were obtained from all participants.

### Procedures for immunohistochemistry (IHC) staining

Tumor and/or normal tissue specimens from the patients were obtained at TURBT and/or cystectomy. Tissue microarrays were constructed by harvesting 2 mm tissue cores (or 1mm tissue cores when only small samples were available) from FFPE tumor or normal tissue samples of BCG-resistant patients. For IHC, the microarray sections (4-μm-thick) were mounted on glass slides, heat-treated for 15 min, and then incubated with rabbit mAb against PD-L1 (clone E1L3N; Cell Signaling Technology, Danvers, MA), mouse mAb against PD-L2 (clone MIH18; eBioscience, San Diego, CA), rabbit polyclonal Ab against CD8 (Abcam, Tokyo, Japan), rabbit mAb against FOXP3 (clone SP97; Abcam), or rabbit polyclonal Ab against CD69 (Abcam) for 30 min, followed by their corresponding secondary antibodies for 30 minutes, with the use of HISTOSTAINER (Nichirei Biosciences Inc., Tokyo, Japan). This automated system used 3, 3′ diaminobenzidine (DAB) as the chromogen (Nichirei Biosciences Inc.). FFPE tissue samples from recurrence-free patients were also sectioned and stained with anti-PD-L1 antibody (E1L3N). PD-L1- or PD-L2-overexpressing HEK293 cells (ATCC, Manassas, VA) that were transiently transfected with PD-L1 or PD-L2 cDNA were prepared as a positive control for staining of each antibody (Supplementary Figure 1).

### Evaluation of IHC staining

For evaluation of PD-L1 and PD-L2 expression, intensity score (IS) and proportion score (PS) were evaluated according to the Allred scoring system [24], and the sums of IS and PS were calculated for each sample. Staining intensity was categorized into 0 (negative), 1 (weak), 2 (intermediate), and 3 (strong), and the strongest intensity in each sample was selected as IS of the sample (Figure 1A and 1B). Staining proportion was calculated as the number of stained cells per 500 cells, and PS was given as follows: 0 (0), 1 (0 - 1/100 cells), 2 (1/100 cells - 1/10 cells), 3 (1/10 cells - 1/3 cells), 4 (1/3 cells - 2/3 cells), and 5 (> 2/3 cells).

Tumor tissues were evaluated for calculation of IS and PS in tumor cells or tumor-infiltrating inflammatory cells. In addition, non-tumor normal tissues were assessed for calculation of IS and PS in inflammatory cells. In BCG-resistant patients, tumor cells and tumor-infiltrating inflammatory cells within tumor tissues and inflammatory cells within non-tumor normal tissues before and after BCG were assessed. In recurrence-free patients, tumor cells and tumor-infiltrating inflammatory cells within tumor tissues were evaluated only before BCG, while inflammatory cells within non-tumor normal tissues were evaluated both before and after BCG.

For evaluation of cytotoxic T cells, the numbers of CD8^+^ cells were counted within tumor or normal tissues, and their density (cells/mm^2^) was assessed (Figure 3A). As shown in Figure 3B, since the numbers of CD8^+^ T cells that formed focal CD8^+^ T cell aggregates within tissues were difficult to count, they were excluded from determination of CD8^+^ T cell density. Similarly, the numbers of FOXP3^+^ Treg cells and CD69^+^ activated lymphocuytes were counted within tumor or non-tumor normal tissues, and their density (cells/mm^2^) was assessed.

Stained sections were evaluated by at least two observers independently. If the evaluations were different between them, the sections were reviewed jointly, and consented results were obtained.

### Statistical analyses

The sample size of this study was small, because the numbers of NMIBC patients whose pathological specimens were available before and after BCG had been limited. Therefore, we performed exploratory statistical analysis in this study. Correlations of PD-L1 expression among tumor cells and inflammatory cells were analyzed using the Spearman’s rank correlation coefficient. The Wilcoxon test was used for comparison of PD-L1 or PD-L2 expression and CD8^+^, FOXP3^+^, or CD69^+^cell density between before and after BCG treatment. All tests were two-sided, and differences were considered statistically significant at p < 0.05. Statistical analyses were performed by using the JMP pro 12 statistical software (SAS Institute, Cary, NC). The box-and-whisker plots were drawn using R (version 3.3.3; The R Foundation, Vienna, Austria). The beeswarm package (The R Foundation) was used for bee swarm plots.

## SUPPLEMENTARY MATERIALS FIGURE



## Abbreviations

BCG: Bacillus Calmette-Guerin
CIS: carcinoma in situ
FFPE: formalin-fixed, paraffin-embedded
IHC: immunohistochemistry
IS: intensity score
mAb: monoclonal antibody
NMIBC: non-muscle-invasive bladder cancer
PD-1: programmed death 1
PD-L1: programmed death-ligand 1
PD-L2: programmed death-ligand 2
PS: proportion score
Treg: regulatory T
TURBT: transurethral resection of bladder tumor
