# Supplementary material for: Enhanced expression of PD-L1 in non-muscle-invasive bladder cancer after treatment with Bacillus Calmette-Guerin
Source: Oncotarget. 2018 Sep 25;9(75):34066–78. doi: 10.18632/oncotarget.26122 (PMC6183350; doi:10.18632/oncotarget.26122)
Supplement: Supplementary file 1 [file oncotarget-09-34066-s001.pdf]

Enhanced expression of PD-L1 in non-muscle-invasive bladder cancer after treatment with Bacillus Calmette-Guerin

SUPPLEMENTARY MATERIALS

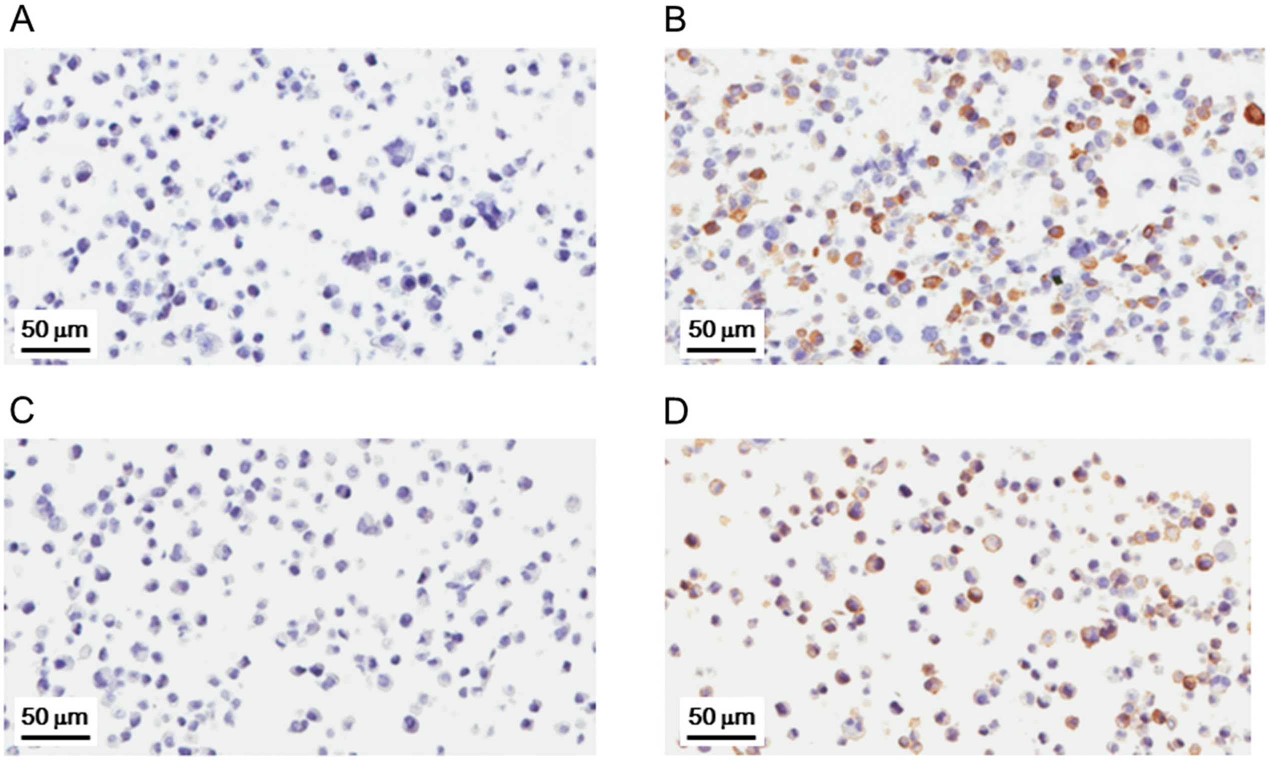

Supplementary Figure 1: PD-L1 and PD-L2 staining in positive control cells. PD-L1 staining in HEK293 cells transiently transfected without **(A)** or with **(B)** PD-L1 cDNA (positive control). PD-L2 staining in HEK293 cells transiently transfected without **(C)** or with **(D)** PD-L2 cDNA (positive control).
